# EEG Channel Selection Using Multiobjective Cuckoo Search for Person Identification as Protection System in Healthcare Applications

**DOI:** 10.1155/2022/5974634

**Published:** 2022-01-12

**Authors:** Zaid Abdi Alkareem Alyasseri, Osama Ahmad Alomari, Mohammed Azmi Al-Betar, Mohammed A. Awadallah, Karrar Hameed Abdulkareem, Mazin Abed Mohammed, Seifedine Kadry, V. Rajinikanth, Seungmin Rho

**Affiliations:** ^1^ITRDC, University of Kufa, P.O. Box 21, Najaf, Iraq; ^2^Center for Artificial Intelligence Technology, Faculty of Information Science and Technology, Universiti Kebangsaan Malaysia, 43600 Bangi, Selangor, Malaysia; ^3^MLALP Research Group, University of Sharjah, Sharjah, UAE; ^4^Artificial Intelligence Research Center (AIRC), College of Engineering and Information Technology, Ajman University, Ajman, UAE; ^5^Department of Information Technology, Al-Huson University College, Al-Balqa Applied University, P.O. Box 50, Al-Huson, Irbid, Jordan; ^6^Department of Computer Science, Al-Aqsa University, P.O. Box 4051, Gaza, State of Palestine; ^7^Artificial Intelligence Research Center (AIRC), Ajman University, Ajman, UAE; ^8^College of Agriculture, Al-Muthanna University, Samawah 66001, Iraq; ^9^College of Computer Science and Information Technology, University of Anbar, Anbar 31001, Iraq; ^10^Department of Applied Data Science, Noroff University College, 4608 Kristiansand, Norway; ^11^Department of Electronics and Instrumentation Engineering, St. Joseph's College of Engineering, Chennai 600119, India; ^12^Department of Industrial Security, Chung-Ang University, Seoul, Republic of Korea

## Abstract

Recently, the electroencephalogram (EEG) signal presents an excellent potential for a new person identification technique. Several studies defined the EEG with unique features, universality, and natural robustness to be used as a new track to prevent spoofing attacks. The EEG signals are a visual recording of the brain's electrical activities, measured by placing electrodes (channels) in various scalp positions. However, traditional EEG-based systems lead to high complexity with many channels, and some channels have critical information for the identification system while others do not. Several studies have proposed a single objective to address the EEG channel for person identification. Unfortunately, these studies only focused on increasing the accuracy rate without balancing the accuracy and the total number of selected EEG channels. The novelty of this paper is to propose a multiobjective binary version of the cuckoo search algorithm (MOBCS-KNN) to find optimal EEG channel selections for person identification. The proposed method (MOBCS-KNN) used a weighted sum technique to implement a multiobjective approach. In addition, a KNN classifier for EEG-based biometric person identification is used. It is worth mentioning that this is the initial investigation of using a multiobjective technique with EEG channel selection problem. A standard EEG motor imagery dataset is used to evaluate the performance of the MOBCS-KNN. The experiments show that the MOBCS-KNN obtained accuracy of 93.86% using only 24 sensors with AR20 autoregressive coefficients. Another critical point is that the MOBCS-KNN finds channels not too close to each other to capture relevant information from all over the head. In conclusion, the MOBCS-KNN algorithm achieves the best results compared with metaheuristic algorithms. Finally, the recommended approach can draw future directions to be applied to different research areas.

## 1. Introduction

Over many years, our universe has transferred to a digital community in which each person is living with a particular digital identifier. Indeed, there are several kinds of identifiers, such as identification cards and passwords. Meanwhile, they can be easily circumvented, stolen, and forgotten [1]. Therefore, personal behavior or characteristics can be used to strengthen identification systems. Such techniques, the so-called biometrics, make use of several pieces of in-person information to allow more robust identification systems, such as face and voice recognition, fingerprint information, and iris data [2]. The motivation of using EEG and body sensors in healthcare systems has been an interesting area for many researchers [3–5].

On the other hand, the widespread and influential deployment of biometric systems leads to a new challenge, which is called “spoofing” [1, 6–8]. Such type of attack is classified as the most dangerous in security systems since it is designed to break the biometric systems' security, thus allowing unwarranted persons to get admission to the system [2].

In real life, there have already been several spoofing attacks on the biometrics systems, such as face spoofing (printed photos and 3D mask attack [9–12]), fake fingerprints (gummy fingers), finger-vein systems fooled through a piece of paper [13], iris recognition systems fooled by an eyeball opposite to the scanner of iris, and voice recognition fooled through replaying a voice recording opposite to the recognition system speaker [14]. Therefore, people are looking for biometric authentication systems that can grant access to a person based on invisible characteristics, thus becoming harder to be attacked by an external threat. In this context, one shall refer to user authentication based on brain signals, which can be captured by the well-known electroencephalogram (EEG) exam [15].

The EEG signals appear as a great alternative for designing new biometric systems since several studies showed that such information presents uniqueness features, universality, and natural robustness to spoofing attacks [1, 16]. These signals represent the graphical recording of the brain electrical activity, which can be measured by placing electrodes (sensors) in different positions of the scalp [1, 17–20]. EEG channel selection is totally dependent on the characteristics of the EEG signal, where the most informative features that provide the highest accuracy rate from the channel selection shall be determined.

Multiobjective optimization algorithms have been applied in different methodological phases and criteria [21, 22]. In a multiobjective formulation of the feature selection problem, the possible features define the vector of decision variables. The space of features in many classification problems is usually very large [23]. In [24, 25], the authors proposed new methods for feature selection, based on a multiobjective evolutionary algorithm, which managed to select a small subset of features, trying to avoid the overfitting problems and reduce classification problems in high-dimensional feature space. In the same direction, the feature selection problem has been also solved by an embedded multiobjective genetic optimization procedure, subject to the simultaneous minimization of the misclassification ratio and number of selected attributes [26, 27]. Multiobjective optimization algorithms have been also used in the deionizing stage of EEG signal. In [28], an approach has been adopted in the signal deionizing stage to model decomposition and present two metrics to quantify the amount of EEG information lost during the cleaning process. Furthermore, in [7], the authors proposed a novel method for extracting unique features from the original EEG signals using the multiobjective flower pollination algorithm and the wavelet transform.

One of the significant challenges concerning EEG-based user identification technique is signal acquisition, which is performed by placing several electrodes (sensors) on a person's head. As a drawback, such a process is usually uncomfortable since it requires good knowledge to place the sensors in their correct positions. Additionally, some questions must be considered such as the following: “Is it really necessary to put all these electrodes on a persons' head? If not, can we identify the most relevant ones for user identification and then use a smaller number of sensors?” The above questions motivated our attempts to model the EEG channel selection as an optimization problem. In this work, we aim at learning the most important EEG channels by proposing a hybrid approach composed of the cuckoo search algorithm (CSA) [29], hereinafter called “MOCS-EEG.” The classification step is performed by KNN classifier. The main contributions of this paper are threefold:

1. To evaluate binary cuckoo search algorithm (BCSA) for EEG-based person identification
2. To model the problem of EEG channel selection as an evolutionary-based optimization task
3. To propose a multiobjective technique combined with the KNN classifier for EEG-based biometric person identification

We use evolutionary optimization algorithms for the EEG channel selection due to their efficiency when solving challenging real-world problems and their simplicity. The performance of the proposed method (MOCS-KNN) is compared with other seven multiobjective metaheuristic algorithms (binary PSO (MOPSO-KNN), binary GWO (MOGWO-KNN), binary FFA (MOFFA-KNN), binary BAT (MOBAT-KNN), binary MVO (MOMVO-KNN), binary WOA (MOWOA-KNN), and binary MFO (MOMFO-KNN)). The comparison includes five measures, which are accuracy ratio (EEG_ACC_), channels selected (EEG_Len_), Specificity (EEG_Precision_), F-score (EEG_Fscore_), and Recall (EEG_Recall_). The results show that the proposed technique (MOCS-KNN) is able to outperform other metaheuristic algorithms in almost all results produced.

The remainder of this paper is organized as follows: the related works about a number of EEG-based identification techniques are reviewed in Section 2. A discussion about cuckoo search algorithm is presented in Section 3. The proposed method is provided in Section 4. Results are discussed in Section 5, and Section 6 provides conclusions and future work.

## 2. Related Works

This section presents an overview of previous studies related to using optimization algorithms in the BCI applications. Several works have been proposed based on optimization algorithms for tackling issues relevant to person identification case study. According to [1, 30], the number of selected channels can effect positively the accuracy of classification task. The authors especially addressed the problem of reducing the number of required sensors while maintaining a comparable performance. They have achieved significant results by obtaining very good person identification rates using much fewer channels. In [1], the authors proposed a binary version of the flower pollination algorithm under different transfer functions to select the best subset of channels that maximizes the accuracy. In [31], the authors proposed a genetic algorithm to reduce the number of necessary electrodes for measurements by EEG devices. The results were encouraging, and it was possible to accurately identify a subject using about 10 out of 64 electrodes. The Authors in [16] proposed hybrid optimization techniques based on binary flower pollination algorithm (FPA) and *β*-hill climbing (called FPA *β*-hc) for selecting the most relative EEG channels (i.e., features) that come up with efficient accuracy rate of personal identification. The proposed method is able to identify persons with high Acc, Sen, *F*_*s*, and Spe and less number of channels selected. However, these studies have concentrated only on the number of channels as baseline for optimization process.

In [7], the authors have proposed novel method for EEG signal denoising based on the multiobjective flower pollination algorithm. They designed a multiobjective function that considers a balance between reducing the EEG noise and keeping its signal energy. In [32], the authors proposed multiobjective optimization method for optimal electroencephalographic (EEG) channel selection to provide access to subjects with permission in a system by detecting intruders and identifying the subject. The optimization process was performed by the nondominated sorting genetic algorithm (NSGA). The optimization process consists of finding the best nu and gamma for the SVM with the RBF kernel to increase the TAR, TRR, and accuracy of subject identification or maintain them as high as possible for previous configurations, while using the smallest number of possible EEG channels. However, the optimization process is restricted within SVM hyperparameters, and it is hard to generalize for another study, especially when using different classifiers where each classifier has its own characteristics and hyperparameters.

To summarize, for person identification within optimization algorithms, two schemes have been observed: first, optimization scheme based on single objective criteria, mainly channel selection; second, optimization scheme based multiobjective criteria such as channel selection, EEG noise, and classifiers hyperparameters. Bioinformatics applications frequently involve classification problems that require improving the learning accuracy [33]. According to [32], certain aspects need to be analyzed and improved before reaching an industrial level application of new biometric systems. One is person identification, which is an essential security layer in any secure system. This is also important for the development of portable low-density EEG devices that retain similar accuracy to high-density EEG. Thus, the accuracy of person identification is very important aspect. To the best of our knowledge, on the one hand, this is the first study to present multiobjective optimization based on the number of channels and the classification accuracy weights as baseline for optimization process. On the other hand, this is the first study to implement and test eight optimization algorithms in order to generalize the best algorithm that can be adopted for the person identification task.

## 3. Cuckoo Search Algorithm

Cuckoo search (CS) algorithm is a natural-based swarm-intelligence metaheuristic proposed by Yang and Deb [34] to imitate the behavior of cuckoo birds in the reproduction process. It simulates the way of cuckoo bird when laying its fertilized eggs in other bird's nest where its children are looked after by proxy parents. Cuckoo bird may also remove the original nest eggs to improve the hatching chance of their eggs. When the proxy parents discover that their foreign eggs do not belong to them, they either throw them out of the nest or abandon the nest. The process of the cuckoo egg reproductions is modeled as an optimization algorithm to formulate CS. Three assumptions are adopted to formulate CS algorithm in optimization context.

- Each cuckoo chooses only one nest to lay one egg in that nest.
- The high quality egg in the best nest is marked to be used in the next generations.
- Since the number of hosting nests is predetermined in advance, the host cuckoo can discover that the eggs in the nest are not its own eggs with a probability of *p*_*a*_ where *p*_*a*_ ∈ (0,1). In this case, the host birds either throw the foreign eggs or abandon the nest and rebuild another one in different place.

**Figure 1:**
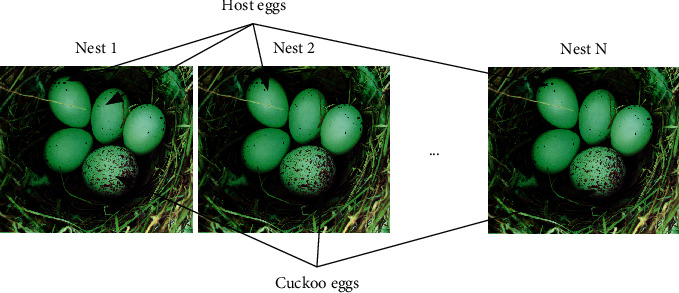
The nests with cuckoo eggs.

In CS algorithm, the eggs in each nest represent the set of solutions while the cuckoo eggs represent the new solution (see Figure 1). The quality of eggs in the nest is the fitness function of that solution. The ultimate aim is to replace the eggs in a nest with potentially better cuckoo eggs. The cuckoo frequently changes its position using Levy flights after leaving nest. The host bird can throw the cuckoo eggs or leave the nest when cuckoo eggs are discovered. The flowchart of the CS algorithm is given in Figure 1. The pseudocode of CS algorithm is given in Algorithm 1.  Table 1 shows the local and global search parameters of CS algorithm. The discussion below provides procedural steps of the CS algorithm.

Step 1: initialize CS algorithm parameters. Initially, the optimization problem is conventionally modeled in terms of objective function as follows:
  (1) $$\begin{array}{c} \begin{array}{cc} \underset{x\in \left[LB,UB\right]}{\min} & f\left(x\right) \end{array}, \end{array}$$
where *f*(**x**) is the objective function to evaluate the quality of the solution **x**=(*x*_1_, *x*_2_,…, *x*_*d*_) of *d* decision variables. Each decision variable *x*_*i*_ ∈ [LB_*i*_, UB_*i*_] in which LB_*i*_ is the lower bound and UB_*i*_ is the upper bound of variable *x*_*i*_, respectively. The parameters of the optimization problem are normally extracted from the datasets. The objective function is used to evaluate the solutions of the problem.
The CS parameters can be divided into two types: (i) algorithmic parameters such as maximum number of iterations and number of nests or population size; (ii) control parameter *p*_*a*_ which is the discovery rate of alien eggs/solutions.
Step 2: initialize the host nest population (HNP). The host nest population is formulated as a matrix HNP of size *N* × *d*, where the *N* is the total number of eggs in the nests and *d* is the solution dimension. Each row in the HNP represents a solution as shown in the following equation:
  (2) $$\begin{array}{c} HNP=\left[\begin{array}{cccc} x_{1}^{1} & x_{2}^{1} & \cdots & x_{d}^{1}\\ x_{1}^{2} & x_{2}^{2} & \cdots & x_{d}^{2}\\ \vdots & \vdots & \cdots & \vdots \\ x_{1}^{N} & x_{2}^{N} & \cdots & x_{d}^{N} \end{array}\right]. \end{array}$$
The objective function value *f*(**x**^*i*^) of each solution **x**^*i*^ is also calculated.
Step 3: this step is also called global random walk. For each solution, **x**^*i*^(*t*) in the **HNP** is updated (i.e., **x**^*i*^(*t*+1)) using Levy flights step as formulated in the following equation:
  (3) $$\begin{array}{c} x^{i}\left(t+1\right)=x^{i}\left(t\right)+\alpha \;⊕\;L\left(s,\lambda \right), \end{array}$$
where
  (4) $$\begin{array}{c} L\left(s,\lambda \right)=\frac{\lambda \Gamma \left(\lambda \right)\sin\left(\pi \lambda /2\right)}{\pi }\frac{1}{s^{1+\lambda }},\;s\geq s_{0}>0, \end{array}$$
where *α* > 0 is the step size scale factor while *s* is the step size. Note that the step size is calculated based on the scale size of the optimization problem on hand [35–37]. The mathematical notation ⊕ refers to pairwise product operation. $\text{L}\overset{´}{e}\text{vy}\left(\lambda \right)$ is the $\text{L}\overset{´}{\text{e}}\text{vy}$ flights and is calculated based on heavy-tailed probability distribution formulated in (3). The random walk is represented in the stochastic equation (4). Γ() is the gamma function.
Step 4: update the host nest population. In order to update the HNP, each solution **x**^*i*^(*t*+1), *i* ∈ (1, ldots, *N*) updated by $\text{L}\overset{´}{\text{e}}\text{vy}$ flights step is compared with another randomly selected solution **x**^*j*^, **x**^*j*^(*t*) ∈ **NHP**. Thereafter, the solution **x**^*j*^(*t*) is replaced by solution **x**^*i*^(*t*+1), if the latter is better.
Step 5: local random probability of *P*_*a*_, each solution **x**^*i*^(*t*+1), *i* ∈ (1, ldots, *N*) in the **HTP**(*t*+1) is check for weather or not abandoned as follows:
  (5) $$\begin{array}{c} x^{i}\left(t+1\right)=x^{i}\left(t\right)+\alpha s⊗H\left(p_{a}-\varepsilon \right)⊗\left(x^{j}\left(t\right)-x^{k}\left(t\right)\right), \end{array}$$
where *x*^*j*^(*t*) and *x*^*k*^(*t*) are two different randomly selected solutions and *H*(*u*) is the Heaviside function. *ε* is a function that generates a random number extracted from a uniform distribution, and *s* is the step size. For more clarifications about the CS algorithm convergence behavior, interesting papers can be referred to [34, 35, 38].
Step 6: stop criterion. Steps 3, 4, and 5 are repeated until the termination criterion; for example, the maximum number of iterations is met.

CS algorithm is successfully applied to solve several optimization problems like traveling salesman problem [39], economic load dispatch [40], face recognition [41], loading pattern optimization [42], data clustering [43], feature selection [44], short-term load forecasting [45], modeling proton exchange membrane fuel cells [46], electric distribution network reconfiguration [47], optimal reactive power scheduling [48], contrast enhancement of gray scale images [49], estimating peak particle velocity in mine blasting [50], systems of nonlinear equations [51], maximizing area coverage in wireless sensor networks [52], job scheduling [53], and others reported in [35, 54, 55].

However, the framework of the CS algorithm is modified and integrated with other components in order to improve its performance. These modifications include CS algorithm with adaptive parameters [56, 57], multiobjective CSA [45, 58], chaotic CSA [43, 59], binary CS algorithm [60, 61], CS algorithm with other metaheuristic algorithms [52, 62, 63], CS algorithm with other components [52, 64], and other improvements of CS algorithm [44, 51, 59, 65].

## 4. Methodology

**Figure 2:**
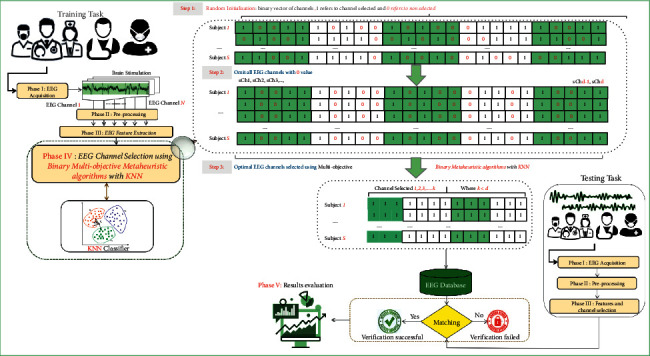
Flowchart of the proposed method (MOCS-KNN).

This section provides a full explanation of the methodology of the proposed MOCS with KNN classifier (MOCS-KNN) to solve EEG channel selection problem. Overall, the methodology has five phases.  Figure 2 shows the flowchart of these phases. Phase I involved EEG signal acquisition task which has been done using 64 electrodes.  Section 5.2 will provide more details about this phase. In Phase II, two conventional filters (band-pass and notch filter) were used to remove unwanted artifacts from the original EEG signal such as those used in [16], and then wavelet was applied to denoise the EEG signal as suggested in [17]. In Phase III, three autoregressive coefficients have been extracted from the denoised EEG signal as feature extraction data, that is, AR5, AR10, and AR20, which are suggested by Rodrigues et al. [1].

Phase IV is the main contribution of this work where a multiobjective cuckoo search algorithm for EEG channel selection is proposed. The following subsections explain in detail the proposed method.

### 4.1. Formulation of Multiobjective Approach

In this work, we used a weight sum approach for implementing multiobjective optimization as suggested by [66]. In the weighted sum approach, the weighting coefficients consider the preferences of the multiple objectives. Basically, the multiobjective optimization for EEG channel selection can be defined as follows:

(6) $$\begin{array}{c} \text{Maximize Fit}=\sum_{k=1}^{N}W_{k}f_{k}, \end{array}$$

with

(7) $$\begin{array}{c} \sum_{k=1}^{N}W_{k}=1,\;\;W_{k}>0, \end{array}$$

where *N* is the number of objective functions and *W*_*k*_ refers to the nonnegative weights.

(8) $$\begin{array}{c} \text{Maximize Fit}=f_{1}\times W_{1}+f_{2}\times W_{2}, \end{array}$$

where *f*_1 and *f*_2 refer to accuracy measure (12) and number of electrodes selected, respectively. *W*_1_=0.8 and *W*_2_=0.2 refer to the weights of *f*_1 and *f*_2.

### 4.2. Transfer Function

**Figure 3:**
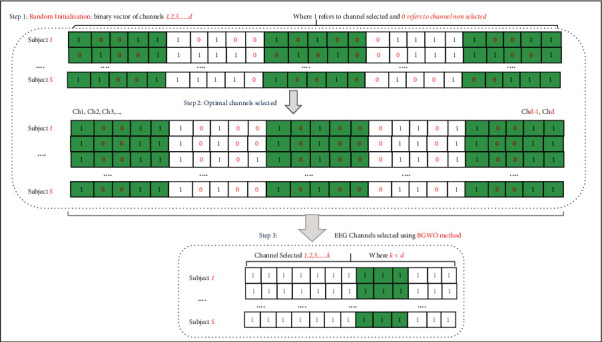
EEG channel selection using the proposed approach MOCS-KNN.

Since the proposed approach was initially designed to handle continuous-valued optimization problems, we need to map each possible solution onto a binary-valued position (i.e., the EEG channel selection problem requires encoding each possible solution as a binary vector, where “0” means the channel will not be used and “1” the opposite situation) [1, 67]. In order to restrict binary solutions only, we need to use the so-called “transfer function” *V*, which is defined as follows:

(9) $$\begin{array}{c} V\left(s_{i}^{t}\right)=\left\{\begin{array}{cc} 1, & \phi >\sigma \left(s_{i}^{t}\right),\\ 0, & \text{otherwise,} \end{array}\right. \end{array}$$

where

(10) $$\begin{array}{c} \sigma \left(s_{i}^{t}\right)=\frac{1}{1+e^{-s_{i}^{t}}}, \end{array}$$

and *ϕ* ~ *U*(0,1).  Figure 3 illustrates how to build a binary vector and to select the optimal EEG subset channels using MOCS-KNN.

There are three steps that must be considered to select the optimal subset of channels. First, random initialization of the binary vector (representing the EEG channels) is conducted, where “1” means that a given channel will be selected and “0” indicates that the channel will not be selected. Later, the MOCS-KNN will start searching for the space to find the optimal subset of channels, i.e., the one that can provide the highest accuracy rate. Finally, we discard all channels with “0” values and keep the remaining ones.

### 4.3. Cuckoo Search Algorithm for EEG Channel Selection

MOCS-KKN is a powerful metaheuristic swarm-based optimization algorithm. MOCS-KKN has a high ability to explore and exploit a particular problem search space using its two control parameters, A and C. In addition, it can explore the search space optimally using its best three solutions. Therefore, MOCS-KKN is adapted for the EEG channel selection problem (MOCS-KKN) in an attempt to find the optimal/near-optimal EEG channel set and achieve the highest accuracy rate. Each solution provided by MOCS-KKN is evaluated based on the objective function (12). The main MOCS-KKN adaptation steps for the EEG channel selection problem are thoroughly discussed below.

**Figure 4:**
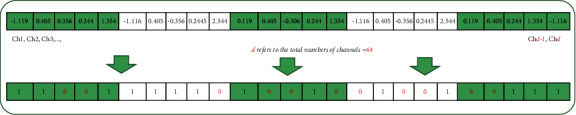
Solution in MOCS-KNN population.

Step 1: initialize MOCS parameters. The first step of adapting MOCS is initializing the EEG channel selection problem and MOCS parameters. The EEG channel selection problem parameters are *CHn*. MOCS parameters are the minimum (LB) and maximum (UB) ranges for the search agent, which are initialized to be 1 and 64, respectively, due to the total number of EEG channels, the number of search agents in the pack (**N**), and the maximum number of iterations (**I**).
Step 2: initialize MOCS population. In this step, all MOCS's solutions are initialized and generated randomly to configure the population. Each solution represents a cuckoo in MOCS and contains the AR feature's coefficient.  Figure 4 presents a solution in MOCS-CS population (P). P contains 30 solutions as shown in the following equation:
  (11) $$\begin{array}{c} P=\left[\begin{array}{cccc} CHn_{1}^{1} & CHn_{2}^{1} & \cdots & CHn_{m}^{1}\\ CHn_{1}^{2} & CHn_{2}^{2} & \cdots & CHn_{m}^{2}\\ \vdots & \vdots & \cdots & \vdots \\ CHn_{1}^{y} & CHn_{2}^{y} & \cdots & CHn_{m}^{y} \end{array}\right]. \end{array}$$
Step 3: objective function evaluation. As mentioned previously, each solution is evaluated based on the objective function in (8). After this evaluation, the best three solutions will be assigned to best cuckoo.
Step 4: update MOCS-KNN population. The population of BMOCS-KNN method will be updated in this step in an attempt to find better solutions and achieve the optimal EEG channel set. This updating can be done using (4). The updating mechanism starts by generating new solutions.
Step 5: check the stop criterion.

Steps 3 and 4 of MOCS-KNN are repeated until the stop criterion is met.

## 5. Result and Discussion

In this section, we discuss the details of the experiments used to assess the robustness of the proposed approach as well as the dataset employed in this work. Parameter setting and experimental setup are later discussed in Section 5.3, while Section 5.4 presents a comparison between the proposed approach, MOCS-KNN, and other metaheuristic algorithms.

### 5.1. The Performance of Traditional EEG Classification Methods

The main purpose of this section is to provide a brief idea about the performance of traditional machine learning classification approach used for EEG-based personal identification problem. The measurements used to evaluate the performance are the classification accuracy and the area under curve (AUC). The results obtained are summarized in Table 2 using three datasets. Several traditional EEG classification methods are experimented with: artificial neural networks (ANN), linear support vector machines, support vector machine with radial basis function (RBF-SVM), *k*-nearest neighbors (*k*-NN), decision tree (J48), optimum-path forest (OPF), Naive Bayes, and linear discriminant analysis (LDA). Based on the results reported, the KNN is able to achieve better classification accuracy for EEG-based personal identification problem. Therefore, the KNN is adopted for MOCS-EEG.

**Figure 5:**
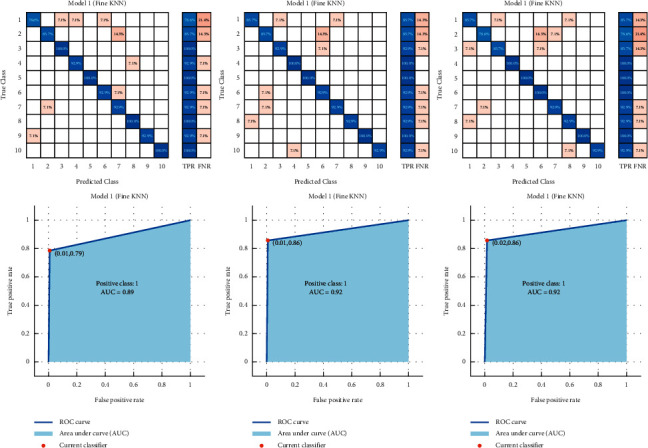
Results of KNN with whole EEG channels.

The area under curve (AUC) measures and confusion matrix are also visualized for KNN results in Figure 5.

### 5.2. Dataset

**Figure 6:**
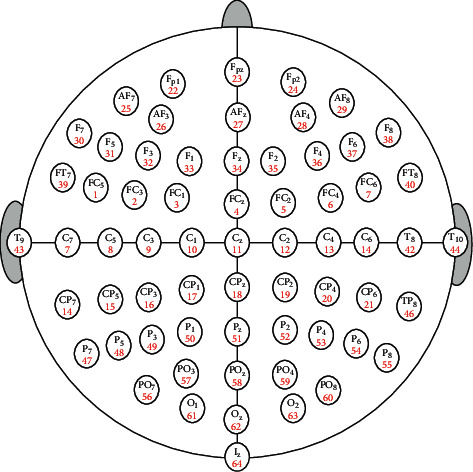
Distribution of the EEG sensors used in this work.

In this work, a standard EEG dataset [68] has been used for evaluating the proposed method (FPA *β*-hc). The brain signals of this dataset are collected from 109 subjects using brain-computer interface software called BCI2000 system [69]. The EEG signals are then acquired from 64 electrodes (i.e., channels), and each subject performs 12 motor/imagery tasks (i.e., 12 records of EEG signals for each subject). Further, AR features are extracted from these 12 recordings with three different number of coefficients: AR5, AR10, and AR20. To reduce the dispersion of the EEG patterns and to quickly process the extracted features, we compute the mean value of each electrode.  Figure 6 shows the distribution of the electrodes of the EEG dataset used in the paper.

### 5.3. Experimental Setup

**Figure 7:**
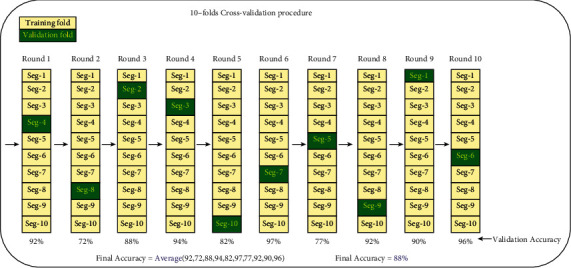
Block diagram of the 10-fold-cross-validation.

In this section, the performance of the proposed channel selection approach and other approaches was evaluated using three EEG signal datasets collected by applying autoregressive (AR) models according to three different coefficients. The solution representation in all channel selection approaches is represented by a vector that consists of a series of 1's and 0's, where “1” means that the channel is selected and “0” means that the channel is ignored. During classification process, the EEG signal data dimension will be reduced and formed solely according to those channels endorsed by channel selection approaches. On the other hand, the channels that were not endorsed will be removed from the original dataset. Afterwards, the classification process is applied here using 10-fold-cross-validation, where the reduced data is iteratively divided into 10 parts, where one part is considered as testing data and the remaining parts as training data. This process is repeated 10 times where the testing part is allocated new samples each time until all samples being covered. The 10-fold-cross-validation process is presented in Figure 7. In this work, the classification accuracy obtained by *k*-nearest neighbors (KNN) and the number of channels are used to design the multiobjective fitness function.

Table 3 shows the parameters used for selected metaheuristic algorithms in this work. With respect to CS, we need to define *β*, which is utilized for the computation of the Levy distribution and *p*_*a*_. *p*_*a*_ stands for the probability of replacing worst nest by new constructed ones.

### 5.4. Comparison between MOCS-KNN and Other Metaheuristic Algorithms

Since the proposed approach is nondeterministic, we computed the mean accuracy rate over 25 runs to avoid biased results. The experiments have been performed using a Lenovo PC, Intel® Core i7 2.59 GHz processor, 12 GB of RAM, and official Windows 10. The performance of the MOCS-KNN is evaluated using five measures: (i) accuracy (EEG_ACC_), (ii) Specificity (EEG_Precision_), (iii) F-score (EEG_Fscore_), (iv) Sensitivity or Recall (EEG_Recall_), and (v) the number of selected channels (EEG_Len_), which can be computed as follows:

(12) $$\begin{array}{c} {\text{EEG}}_{\text{ACC}}=\frac{\text{TA}+\text{TR}}{\text{TA}+\text{FA}+\text{TR}+\text{FR}}\times 100, \end{array}$$

(13) $$\begin{array}{c} {\text{EEG}}_{\text{Recall}}=\frac{\text{TA}}{\text{TA}+\text{FR}}, \end{array}$$

(14) $$\begin{array}{c} {\text{EEG}}_{\text{Precision}}=\frac{\text{TA}}{\text{TA}+\text{FA}}, \end{array}$$

(15) $$\begin{array}{c} {\text{EEG}}_{\text{Fscore}}=2\times \left(\frac{{\text{EEG}}_{\text{Recall}}\;\times {\text{ EEG}}_{\text{Precision}}}{{\text{EEG}}_{\text{Recall}}+{\text{EEG}}_{\text{Precision}}}\right), \end{array}$$

where FR, FA, TA, and TR represent the false rejection, false acceptance, true acceptance, and true rejection, respectively.

**Figure 8:**
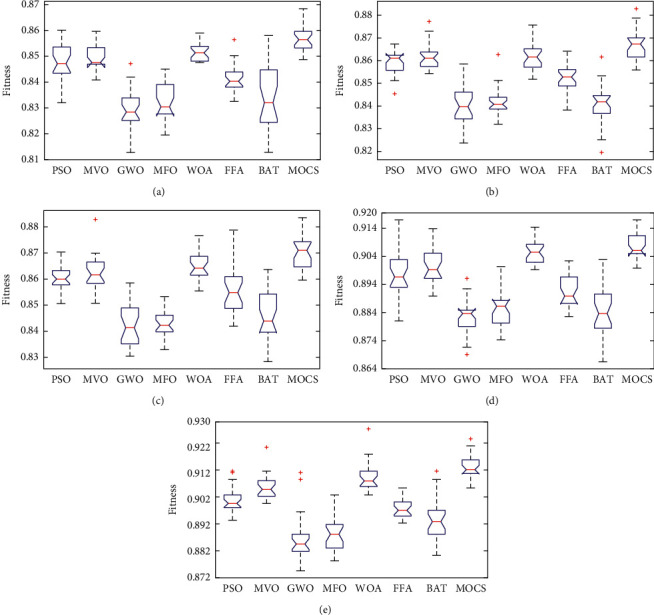
Boxplot of MOCS with other metaheuristic algorithms. (a) Boxplot of AR5. (b) Boxplot of AR10. (c) Boxplot of AR20. (d) Boxplot of TDF. (e) Boxplot of T-FDF.

**Figure 9:**
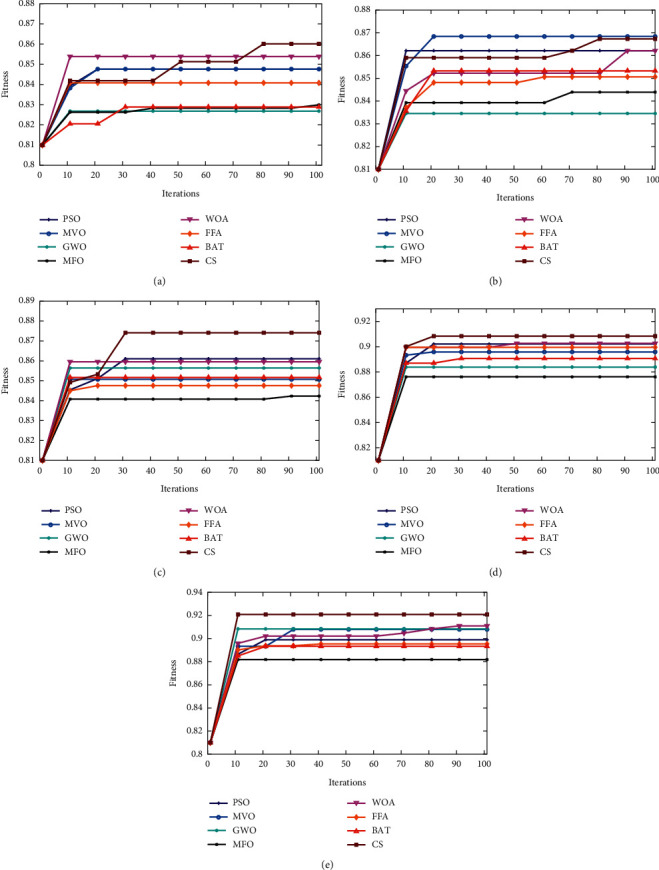
Convergence rate of MOCS with other metaheuristic algorithms. (a) Convergence rate of AR5. (b) Convergence rate of AR10. (c) Convergence rate of AR20. (d) Convergence rate of TDF. (e) Convergence rate of T-FDF.

**Figure 10:**
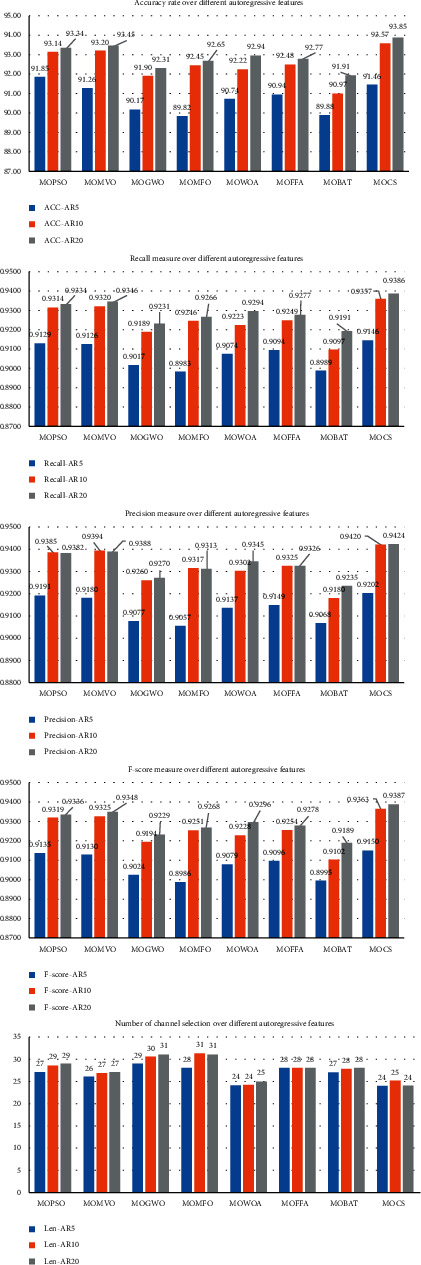
Evaluation measures of MOCS with other metaheuristic algorithms.

Figures 8–10 show the boxplot and convergence rate over 25 runs for selected metaheuristic algorithms during the experimental evaluation using AR5, AR10, and AR20, respectively. The boxplot components are defined as follows: box length illustrates interquartile range, the whiskers indicate the range of the values, the horizontal line in the box indicates the median value, and the outliers are represented by the circles. The boxplots reveal that MOCS-KNN managed to yield highly accurate results. As shown in Figure 8, for AR5 and AR20 datasets, it can be observed that MOCS-KNN shows a superior efficacy in convergence trends compared to other metaheuristic algorithms. For AR10 dataset, MOCS-KNN and MOMVO-KNN show competitive efficacy in the early stage of convergence, but in the later stages, MOCS-KNN surpasses MOMVO-KNN. Overall, MOCS-KNN shows improved convergence learning due to its capability to pave the way for its swarm to achieve the best trajectory leading to global optimal solution by avoiding stagnation drawbacks.

To further validate the results obtained by MOCS-KNN and other methods, Wilcoxon signed-rank statistical test [70] is adopted in this study to show if there is statistically significant difference between these methods. In Table 4, *Z*-value represents standardized test statistics, and *P*-value represents the statistical significance (*P* < 0.05). A *P* value <0.5 means that there is statistical significant difference between paired methods; otherwise, there is no statistical significant difference. From Table 4, it can be observed that MOCS-KNN achieved statistical significant results in all datasets when compared with other methods.

In this section, the performance of the proposed method (MOCS-KNN) is compared with other seven metaheuristic algorithms (PSO algorithm (MOPSO-KNN), GWO algorithm (MOGWO-KNN), binary FFA algorithm (MOFFA-KNN), binary BAT (MOBAT-KNN), MVO (MOMVO-KNN), WOA (MOWOA-KNN), and MFO (MOMFO-KNN)). The comparison includes five measures, which are accuracy ratio (EEG_ACC_), channels selected (EEG_Len_), Specificity (EEG_Precision_), *F*-score (EEG_Fscore_), and Recall (EEG_Recall_).  Table 5 shows the results of proposed technique (MOCS-KNN) with other metaheuristic algorithms using autoregressive three different coefficients. Overall, it is worth mentioning that the MOCS-KNN obtained the best results on all measurements for all datasets. To be more precise, in terms of number of channels, the results of MOCS-KNN and MOWOA-KNN are equal, where MOCS-KNN obtained the minimum number of channels in AR20 (24), while MOWOA-KNN obtained the minimum number of channels in AR10 (25), and both methods minimize the number of channels in AR5 to the least length (24). To gain a clear overview of the performance of MOCS-KNN and other methods in all measurements, the summation of ranks is applied in AR5, AR10, and AR20 datasets for all measurements as shown in Table 6. To elaborate the summation of ranks procedure, the method that achieved the best result will be given a rank of “1,” the second best method will be given a rank of “2,” etc. The summation of ranks in the last row of Table 6 represents the sum of ranks of each method with the corresponding datasets. The bold font highlights the best result. The results suggest that MOCS-KNN achieved the best performance in all evaluation measurements, followed by MOMVO, MOPSO, MOWOA, MOFFA, MOMFO, and MOGWO.

## 6. Conclusion and Future Work

In this work, we proposed a binary version of multiobjective approach using several metaheuristic algorithms with the aim of addressing the challenge of channel selection in EEG-based biometric person identification. The main purpose of this work is to demonstrate that not all available EEG channels need to be used to achieve high accuracy rate. Therefore, we introduce for modeling the problem of channel selection as an optimization issue, where the channel subset which optimizes the recognition ratio over a validation set is utilized as the fitness function.

The outcomes of experiments showed that the introduced method outperformed several metaheuristic algorithms and the one proposed by Rodrigues et al. [1]. It is worth noting that, while retaining high accuracy rates, the number of sensors has been lessened by half. Additionally, the outcomes displayed a positive correlation between the number of features obtained from the EEG signal and the accuracy ratio; i.e., further features lead to higher accuracy rates. Such finding suggests that the proposed algorithm has the potential to remove duplicate and undesirable features whereas retaining specific features.

Regarding the future work, we intend to evaluate selected metaheuristic algorithms over different features, such as time- and frequency-domain information, to improve the overall identification performance while selecting fewer channels.

## Figures and Tables

**Algorithm 1:**
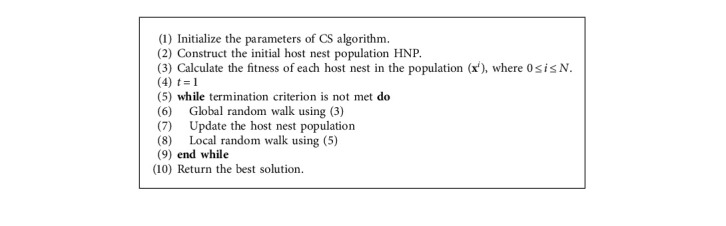
The CS algorithm pseudocode.

**Table 1:** Local and global search parameters of CS algorithm.

Parameter	Description
*x*_*i*_^*t*+1^	The next position
*x*_*k*_^*t*^	The current position selected randomly at the position *k*
*x*_*j*_^*t*^	The current position selected randomly at the position *j*
*α*	Positive step size scaling factor
*s*	Step size
⊗	Entrywise product of two vectors
*H*	Heaviside function
*p*_*a*_	Used to switch between local and global random walks
*ε*	Random number from uniform distribution
*L*(*s*, *λ*)	$\text{L}\overset{´}{\text{e}}\text{vy}$ distribution, used to define the step size of random walk

**Table 2:** KNN results.

Features	Number of channels	Accuracy (%)
AR5	64	87
AR10	64	91
AR20	64	92

**Table 3:** Metaheuristic parameters.

Approach	Parameters
MOCS	*β*=1.5, *P*_*a*_=0.25
MOPSO	*c*1=*c*2=2
MOMVO	WEPMax=1, WEPMin=0.2
MOBAT	*A*=0.5, *r*=0.5
MOFFA	*α*=0.5, *γ*=1, *β*_0_=0.2
MOMOF	—
MOGWO	—
MOWOA	*a* = [2, 0], *b* = 1

**Table 4:** Wilcoxon signed-rank test of MOCS-KNN and other metaheuristic algorithms.

Dataset	Metaheuristic	*Z*-value	*P* value	MOCS-KNN
AR5	MOPSO-KNN	−4.3724	<0.00001	Significant
	MOGWO-KNN	−4.3724	<0.00001	Significant
	MOFFA-KNN	−4.3724	<0.00001	Significant
	MOBAT-KNN	−4.2109	<0.00001	Significant
	MOMVO-KNN	−4.2109	<0.00001	Significant
	MOWOA-KNN	−4.3724	<0.00001	Significant
	MOMFO-KNN	−4.3724	<0.00001	Significant

AR10	MOPSO-KNN	−4.3724	<0.00001	Significant
	MOGWO-KNN	−4.3724	<0.00001	Significant
	MOFFA-KNN	−4.3724	<0.00001	Significant
	MOBAT-KNN	−4.1302	<0.00001	Significant
	MOMVO-KNN	−4.3724	<0.00001	Significant
	MOWOA-KNN	−4.3724	<0.00001	Significant
	MOMFO-KNN	−4.3724	<0.00001	Significant

AR20	MOPSO-KNN	−2.758	0.00578	Significant
	MOGWO-KNN	−4.3455	<0.00001	Significant
	MOFFA-KNN	−4.3455	<0.00001	Significant
	MOBAT-KNN	−4.3455	<0.00001	Significant
	MOMVO-KNN	−2.166	0.03	Significant
	MOWOA-KNN	−4.184	<0.00001	Significant
	MOMFO-KNN	−4.0226	<0.00001	Significant

TDF	MOPSO-KNN	−3.7132	0.0002	Significant
	MOGWO-KNN	−3.1143	0.00188	Significant
	MOFFA-KNN	−4.3724	<0.00001	Significant
	MOBAT-KNN	−4.3724	<0.00001	Significant
	MOMVO-KNN	−3.1143	0.00188	Significant
	MOWOA-KNN	−1.9238	0.05486	Nonsignificant
	MOMFO-KNN	−4.3724	<0.00001	Significant

T-FDF	MOPSO-KNN	−4.1973	<0.00001	Significant
	MOGWO-KNN	−4.3724	<0.00001	Significant
	MOFFA-KNN	−4.3724	<0.00001	Significant
	MOBAT-KNN	−4.332	<0.00001	Significant
	MOMVO-KNN	−3.7714	0.00016	Significant
	MOWOA-KNN	−2.5571	0.01046	Significant
	MOMFO-KNN	−4.3724	<0.00001	Significant

**Table 5:** Comparison of multiobjective cuckoo search algorithm with other metaheuristic algorithms.

Algorithms	Measures	AR5	AR10	AR20	TDF	T-FDF
MOPSO-KNN	EEG_Fit_	0.8483	0.8593	0.8604	0.8988	0.9008
	EEG_ACC_	91.29	93.14	93.34	0.9671	0.9714
	EEG_Len_	27	29	29	24	24
	EEG_Precision_	0.9129	0.9314	0.9334	0.9722	0.9764
	EEG_Recall_	0.9191	0.9385	0.9382	0.9671	**0.9764**
	EEG_Fscore_	0.9135	0.9319	0.9336	0.9673	0.9717

MOCS − KNN	EEG_Fit_	**0.8556**	**0.8665**	**0.8705**	**0.9078**	**0.9133**
	EEG_ACC_	**91.46**	**93.57**	**93.86**	0.9668	0.9714
	EEG_Len_	**24**	25	**24**	**21**	**20**
	EEG_Precision_	**0.9146**	**0.9357**	**0.9386**	0.9716	0.9763
	EEG_Recall_	**0.9202**	**0.9420**	**0.9424**	0.9668	0.9714
	EEG_Fscore_	**0.9150**	**0.9363**	**0.9387**	0.9670	0.9716

MOMVO − KNN	EEG_Fit_	0.8492	0.8619	0.8627	0.9011	0.9058
	EEG_ACC_	91.26	93.20	93.46	**0.968**	0.9734
	EEG_Len_	26	27	27	23	23
	EEG_Precision_	0.9126	0.9320	0.9346	**0.9730**	**0.9779**
	EEG_Recall_	0.9180	0.9394	0.9388	**0.968**	0.9734
	EEG_Fscore_	0.9130	0.9325	0.9348	**0.9680**	**0.9737**

MOBAT − KNN	EEG_Fit_	0.8346	0.8408	0.8469	0.8849	0.8931
	EEG_ACC_	89.89	90.97	91.91	0.9568	0.9665
	EEG_Len_	27	28	28	26	26
	EEG_Precision_	0.8989	0.9097	0.9191	0.9635	0.9722
	EEG_Recall_	0.9068	0.9180	0.9235	0.9568	0.9665
	EEG_Fscore_	0.8995	0.9102	0.9189	0.9569	0.9668

MOFFA − KNN	EEG_Fit_	0.8412	0.8519	0.8559	0.8920	0.8975
	EEG_ACC_	90.94	92.49	92.77	0.9642	0.9691
	EEG_Len_	28	28	28	25	25
	EEG_Precision_	0.9094	0.9249	0.9277	0.9702	0.9746
	EEG_Recall_	0.9149	0.9325	0.9326	0.9642	0.9691
	EEG_Fscore_	0.9096	0.9254	0.9278	0.9645	0.9694

MOMFO-KNN	EEG_Fit_	0.8326	0.8419	0.8429	0.8859	0.8880
	EEG_ACC_	89.82	92.46	92.66	0.9605	0.964
	EEG_Len_	28	31	31	26	27
	EEG_Precision_	0.8983	0.9246	0.9266	0.9672	0.9707
	EEG_Recall_	0.9057	0.9317	0.9313	0.9605	0.964
	EEG_Fscore_	0.8986	0.9251	0.9268	0.9607	0.9643

MOGWO-KNNN	EEG_Fit_	0.8294	0.8398	0.8425	0.8825	0.8869
	EEG_ACC_	90.17	91.90	92.31	0.9577	0.9628
	EEG_Len_	29	30	31	26.76	26.68
	EEG_Precision_	0.9017	0.9189	0.9231	0.9647	0.9688
	EEG_Recall_	0.9077	0.9260	0.9270	0.9577	0.9628
	EEG_Fscore_	0.9024	0.9194	0.9229	0.9577	0.9630

MOWOA-KNN	EEG_Fit_	0.8517	0.8618	0.8652	0.9054	0.9092
	EEG_ACC_	90.74	92.23	92.94	0.9674	0.9697
	EEG_Len_	**24**	**24**	25	21.92	21.28
	EEG_Precision_	0.9074	0.9223	0.9294	0.9724	0.9747
	EEG_Recall_	0.9137	0.9302	0.9345	0.9674	0.9697
	EEG_Fscore_	0.9079	0.9228	0.9296	0.9675	0.9699

Bold values indicate best results.

**Table 6:** Sum of ranks of autoregressive features using several evaluation measures.

Measures	MOPSO	MOMVO	MOGWO	MOMFO	MOWOA	MOFFA	MOBAT	MOCS
ACC-AR5	2	3	6	8	5	4	7	**1**
ACC-AR10	3	2	7	5	6	4	8	**1**
ACC-AR20	3	2	7	6	4	5	8	**1**
ACC-TDF	4	3	8	6	2	5	7	**1**
Len-T-FDF	4	3	8	7	2	5	6	**1**
Len-AR5	3	2	5	4	**1**	4	3	**1**
Len-AR10	5	3	6	7	**1**	4	4	2
Len-AR20	5	3	6	6	2	4	4	**1**
Len-TDF	4	3	7	6	2	5	6	**1**
Len-T-FDF	4	3	7	8	2	5	6	**1**
Recall-AR5	2	3	4	8	6	5	7	**1**
Recall-AR10	3	2	7	4	6	5	8	**1**
Recall-AR20	3	2	7	6	4	5	8	**1**
Recall-TDF	3	**1**	7	6	2	5	8	4
Recall-T-FDF	**1**	2	8	7	4	5	6	3
Precision-AR5	2	3	6	8	5	4	7	**1**
Precision-AR10	3	2	7	6	4	5	8	**1**
Precision-AR20	3	2	7	6	4	5	8	**1**
Precision-TDF	3	**1**	7	6	2	5	8	4
Precision-T-FDF	2	**1**	8	7	4	5	6	3
F-score-AR5	2	3	6	8	5	4	7	**1**
F-score-AR10	3	2	7	5	6	4	8	**1**
F-score-AR20	3	2	7	6	4	5	8	**1**
F-score-TDF	3	**1**	7	6	2	5	8	4
F-score-T-FDF	2	**1**	8	7	4	5	6	3
Summation of ranks	75	55	170	159	89	117	170	**41**

Bold values indicate best results.

## Data Availability

The dataset was taken from https://www.physionet.org/.
